# Dietary Intake patterns in women with GDM and Non-GDM: A comparative study

**DOI:** 10.12669/pjms.38.7.5889

**Published:** 2022

**Authors:** Shabnam Nadeem, Aisha Khatoon, Shaista Rashid, Fauzia Ali

**Affiliations:** 1 Dr. Shabnam Nadeem, Associate Professor, Gynae Unit III, Karachi Medical & Dental College, Abbasi Saheed Hospital, Karachi, Pakistan; 2 Prof. Dr. Aisha Khatoon, Gynae Unit III, Karachi Medical & Dental College, Abbasi Saheed Hospital, Karachi, Pakistan; 3 Dr. Shaista Rashid, Associate Professor, Gynae Unit III, Karachi Medical & Dental College, Abbasi Saheed Hospital, Karachi, Pakistan; 4 Dr. Fauzia Ali, Assistant Professor, Gynae Unit I, Karachi Medical & Dental College, Abbasi Saheed Hospital, Karachi, Pakistan

**Keywords:** Dietary intake patterns, GDM, Non-GDM

## Abstract

**Objectives:**

The study aimed to determine dietary Intake patterns in women with GDM and Non-GDM, a comparative study in a tertiary care hospital, Pakistan.

**Methods:**

This comparative cross sectional study was conducted through questionnaire spread over a period of six months of pregnant women visiting to Abbasi Shaheed Hospital for ante-natal visit having 24 to 28 weeks of gestation. With the written consent of the participants dietary intake patterns were assessed in GDM & Non-GDM subjects by a three day 24 hours’ recalls and food frequency questionnaire. A 24-hour dietary recall chart is a dietary assessment tool in which participants were asked to recall all food and drink they have consumed in the last 24 hours. The FFQ (food frequency questionnaire) provide a list of foods and participants were asked how often they eat each item on the list. This FFQ has 70 food items. The food frequency was reported as never, per year, per month, once a week, once and a day. The reported intake of food was converted into nutrients intake (carbohydrate, protein, fat) which was calculated by reported intake frequency of each food multiplied by reported portion size and its respective nutrient composition, summing over all foods by a trained Nutritionist.

**Results:**

A total of 75 participants with GDM, and 75 with Non-GDM were enrolled in this study over a period of six months. It was observed that dietary intake patterns have a significant association with GDM. Those who consume carbohydrate mainly containing diet have likely to have GDM. It has been seen that those who have family history of diabetes are more likely to have GDM. Family dietary patterns can affect risk of GDM. Our study has shown that timings of meals did not find have any significant association with GDM.

**Conclusion:**

Dietary patterns strongly influence the risk of GDM. The most contributing factors to risk of GDM are higher intake of carbohydrate rich diet and lesser consumption of fruits and vegetables.

## INTRODUCTION

Gestational Diabetes Mellitus (GDM is defined as any degree of glucose intolerance first diagnosed in pregnancy.^1^ According to World Health Organization (WHO), the new criteria for the diagnosis of hyperglycemia is based on using one step approach of 75-g 2-hours oral glucose tolerance test (OGTT) with one or more abnormal values.^2^

Inadequately managed GDM is significantly associated with pregnancy complications. Without preventive care almost half of women with gestational diabetes progress to type-2 diabetes and a substantial proportion develop premature cardiovascular diseases within 10 years of child birth.^3^

Globally, the prevalence of GDM is increasing by more than 30% within one or two decades especially in developing countries.^4^ Evidence has suggested that dietary intake before and during pregnancy is associated with the risk of GDM.^5^ High consumption of red meat, processed meat, egg and animal protein, heme iron, fat and cholesterol can increase the GDM risk.^5^ However, increase intakes of fiber, nuts and vegetable derived protein may lower the risk of GDM.^6^ Foods are commonly consumed in combination so dietary patterns analysis is a useful approach to evaluate diet-GDM relationship. Studies of dietary patterns and GDM in Asia remains scarce with conflicting results as dietary intake were influenced by socioeconomic, ethnic factors and cultured values and individual taste of foods.^6^ A study Southern China showed a positive association between seafood and sweet patterns and risk of GDM.^7^ Another study showed an inverse association for seafood and noodle pattern and risk of GDM in a multi – ethnic Asian cohort.^8^

Few studies have been conducted in Pakistan to assess dietary patterns during pregnancy but none has analyzed their association with GDM A study was conducted in Islamabad has shown that medium dietary diversity was observed in 89% of pregnant women.^9^ Another study in Rawalpindi has shown that half of study participants were not taking adequate food.^10^ A study was conducted in Punjab has shown that in adequate ante-natal counseling and affordability of food items were main barrier to take healthy diet during pregnancy.^11^

Lower to middle class group dominate percentage wise in Pakistan’s population. In this group the literacy rate is low and they are unaware of importance of dietary patterns during pregnancy. Besides due to economic strain the population prefer to take carbohydrate rich diet and cannot afford to take food rich in protein, fruits and vegetables. This leads to increase insulin resistance and ultimately results in GDM.

The Abbasi Shaheed Hospital is a tertiary care hospital, where health care providers can provide awareness about importance of dietary intake patterns during pregnancy and its association with GDM. Therefore, a study was planned to assess dietary intake patterns during pregnancy and their association with GDM.

## METHODS

It was a comparative cross sectional study. Ethical approval was obtained from Ethical and Scientific Review Committee of KM&DC (ESRC/KM&DC/049/19, 14-07-2020). The participants included were from all out door women between 18 to 40 years of age, having 24 to 28 weeks of gestation, coming to Abbasi Shaheed Hospital for routine ante-natal visit. An informed written consent was taken from the participants. The GDM subjects recruited from women visiting the ante-natal clinic and OGTT was performed on the basis of any of the risk factors identified at initial ante-natal visit, which includes, BMI >30Kg, previous macrosomic baby≥ 4.5 Kg, previous history of GDM, IUD and family history of diabetes. Non-GDM subjects were randomly selected from women who had no identified risk factors over the same period as the GDM patients by non-convenient sampling technique. Participants with pregnancy having Medical disorders (PIH, Pre-eclampsia, Thyroid disorders with obstetric complications (APH, PPROM, PTL), Pregnancy with febrile medical illnesses (UTI, Enteric, Malaria) and multiple pregnancies were excluded from study.

The sample size was 150 using WHO software. The reference study used, published in JCPSP 2014.^12^ The data was collected through filling of Performa and questionnaires and laboratory results of OGTT. The Performa contains demographic information regarding participant’s age, education, ethnicity, parity, working status and socioeconomic status.

Dietary intake patterns of the participants were assessed by a three day 24 hours’ recalls and food frequency questionnaire conducted by principal investigator or one or two trained doctors under supervision of the principal investigator. A 24-hour dietary recall chart is a structured dietary assessment tool in which participants were asked to recall all food and drink they have consumed in the last 24 hours.^12^ Details might include timing, sources of food, type of food, portion size of food, beverages, any dietary supplement, consumption of food at home and outside from home. The 24 –hour dietary recall is most accurate when administered more than once for each individual so that three day 24 hour recall will be collected .The last two day’s record and Sunday record was obtained at the time of interview.

The FFQ (food frequency questionnaire) provide a list of foods and participants were asked how often they eat each item on the list.^13^ The FFQ can assess dietary intake in a way that is valid, easy and in expensive to administer and can be easily utilized in studies for promoting health and assessing intake. This FFQ has 70 food items. The food frequency was reported as never, per year, per month, once a week, once and a day.

The reported intake of food was analyzed to determine nutrients intake which was calculated by reported intake frequency of each food multiplied by reported portion size and its respective nutrient composition, summing over all foods by a trained Nutritionist. The composition of raw food items was determined from the USDA.^14^ In certain cases, where this information was not available from USDA, other local food composition table was consulted.^15^,^16^

Dietary intake patterns were assessed in GDM & Non-GDM subjects and association was analyzed by SPSS version 17. Continuous variables like age was presented as Mean, Standard Deviation, Median, Range, 95%CI whereas categorical variables like ethnicity, working status, socioeconomic status and dietary intake patterns was presented in frequency and percentages. A Chi-square test applied to evaluate the association of dietary intake with GDM and Non-GDM. A p -value<0.05 was considered as statistically significant.

## RESULTS

A total of 75 participants with GDM, and 75 with Non-GDM were enrolled in this study over a period of six months. The demographic characteristics are shown in Table-I. The mean age of the subjects was 29.02±4.67. Most subjects were highly educated 66(44%) and 13(8%) were illiterate. Majority117 (78.5%) belonged to Urdu speaking ethnicity and 130 (87.0%) were household women. As far as parity was concerned, most of our participants were mother of more than one child 88 (59.0%). Majority of participants were belonging to lower to middle class 110 (73%). Regarding diabetes, 77(47.7%) of participants had family history of diabetes while 54(34.9%) had history of GDM in her previous pregnancies.

**Table I:** Baseline characteristics.

		Number (%)
Education	Illiterate	13 (8.7)
	Primary	25 (16.8)
	Secondary	45 (30.2)
	High education	66 (44.3)
	House hold	130 (87.2)
Working	Part time	16 (10.7)
	Full time worker	3 (2.0)
	Urdu	117 (78.5)
Ethnicity	Sindhi	3 (2.0)
	Pathan	18 (12.1)
	Punjabi	11 (7.4)
	Primi-gravida	53 (35.6)
Parity	Multi-gravida	88 (59.1)
	grand multi gravida	8 (5.4)
	Lower	39 (26.2)
Socioeconomic	Middle	67 (45.0)
	Upper	43 (28.9)
History of GDM in Previous pregnancies		52 (34.9)
Family history of diabetes		71 (47.7)

On evaluating the dietary intake patterns it was evident that carbohydrate was the key nutrient in diet taken by participants of the two groups along with minimal intake of protein and fat in diet. Fig-I

**Fig.1:**
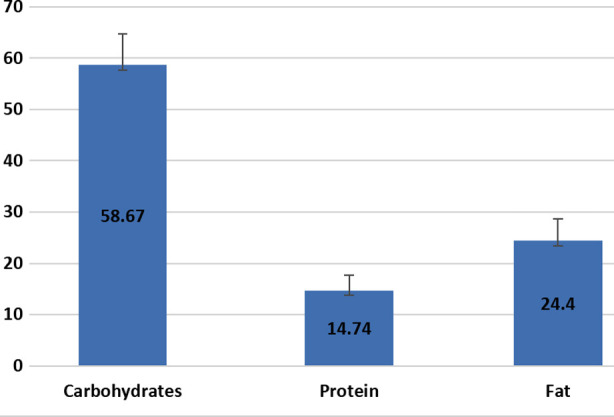
Dietary intake during pregnancy.

Timing of meals was assessed during 24 hours 3- day re-call interview. It showed that almost half 62(41%), 70(47%) of participant did not took breakfast and lunch on time respectively and 128 (85.9%) took dinner on time. As far as Sunday timing was concerned, 118(79.2%) did not take breakfast on time and 98(65.8%) did not took lunch on time.

Association of dietary intake patterns with GDM and Non-GDM is shown in Table-II. It was observed that dietary intake patterns have a significant association with GDM. Those who consume carbohydrate mainly containing diet were likely to have GDM p value=0.006. It was noted that those who have family history of diabetes are more likely to have GDM p value= 0.001. As far as previous history of GDM was concerned those who had GDM in her previous pregnancy36 (48%) were more likely to have GDM in her current pregnancy.

**Table II:** Association of dietary intake and with and without GDM.

		GDM		P-value

		Yes	Non	
Carbohydrates		60.54 ± 5.09	57.67 ± 6.37	0.006*
Protein		14.18 ± 2.20	15.05 ± 3.34	0.090
Fat		23.69 ± 4.18	24.78 ± 4.22	0.134
Family history of diabetes	Yes	54 (72.0%)	17 (23.0%)	0.001*
	No	21 (28.0%)	57 (77.0%)	
History of GDM in previous pregnancies	Yes	36 (48.0%)	16 (21.6%)	0.001*
	No	39 (52.0%)	58 (78.4%)	

* categorical data is presented as frequency (%) and numeric data as mean ± SD.

P-value < 0.05 was considered as statistical significant.

We did not find any significant association of timings of meals with GDM. Table-III has shown that there was no significant association of dietary nutrients intake patterns with demographics (parity, socioeconomics, age, education, and ethnicity).

**Table III:** Association of dietary intake with parity and socioeconomic class

		Mean ± SD	P-value
Carbohydrate	Primi gravida	58.23 ± 6.23	0.47
	Multi gravida	58.78 ± 6.25	
	Grand multi	60.38 ± 2.62	
Protein	Primi gravida	14.63 ± 3.19	0.89
	Multi gravida	14.93 ± 2.98	
	Grand multi	13.50 ± 2.00	
Fat	Primi	24.34 ± 4.33	0.43
	Multi gravida	24.32 ± 4.34	
	Grand multi	25.75 ± 1.67	
Carbohydrate	Lower	58.15 ± 6.53	0.25
	Middle	58.69 ± 6.18	
	Upper	59.12 ± 5.65	
Protein	Lower	14.45 ± 2.59	0.75
	Middle	14.62 ± 2.78	
	Upper	15.21 ± 3.67	
Fat	Lower	25.00 ± 4.96	0.52
	Middle	24.16 ± 4.47	
	Upper	24.23 ± 2.97	

## DISCUSSION

This study assessed the dietary intake patterns during pregnancy and their association with risk of GDM at public sector hospital in Pakistan. The dietary patterns during pregnancy were primarily highlighted in this study. There was no specific dietary pattern observed in our study except that most of our pregnant women consumed carbohydrate rich diet with less intake of protein. The food items observed were mainly chappati, paratha, biryani, bakery items, potato chips and French fries and carbonated drinks.

A food portion with high energy and sugar contents with lower proteins is considered to be a less healthy dietary pattern. This pattern is not observed in western countries earlier. A study by Hoffmann et al.^17^ showed that Brazilian patterns involving intake of rice, pasta combined with beans and beef, egg, chicken and packed fruit juices. Another pattern where eggs, peaches, cereals, fried fish liver, pork meat and fresh fruit juices were mostly consumed was reported in study by Volgyi E et al in Southern United states.^18^ This is mainly due to the differences in culture and lifestyle in western and eastern countries.

Furthermore, in this unhealthy dietary pattern, a substantial relationship was noted with the risk of GDM. Our findings are consistent with study published in Iran showing that unhealthy dietary patterns were strongly associated with risk of GDM.^19^

A significant association was found between consumption of high carbohydrate and low protein (egg, meat, fish) diet patterns and the risk of GDM in our study p value=0.006. Our findings are not consistent with the study conducted among Chinese pregnant women where an inverse relationship was found between consumption of high protein-low starch food and the risk of GDM.^20^ Moreover, these findings are also not consistent with the study conducted in India showing that red meat consumption was associated with an increased risk of GDM.^21^ Fruits/ vegetables are rich in dietary fibers which may decrease insulin resistance. Furthermore, dietary fibers intake can prolong gastric emptying time and slow food digestion and absorption, thus decreasing post prandial plasm glucose levels.^22^ In our study our participants did not like vegetables and majority can’t afford fruits in their diet which may be a contributory factor for risk of GDM. Our findings are consistent with the study conducted in Lahore showing that high carbohydrate intake with low intake of fruits and green leafy vegetables had increased risk of hyperglycemia of pregnancy.^23^ Another study conducted in AKUH has also shown that participants who reduced or stopped the use of fruits during pregnancy were likely to have GDM^24^ but our findings are not consistent with a multiethnic cohort study conducted in Singapore showing that diet rich in vegetable-fruits were associated with low risk of GDM.^8^

There are many factors which are responsible for this unhealthy dietary pattern such as family size, socioeconomic status, family education and cultural reasons, traditional beliefs, liking or disliking of food items.

Genetic factors are important in determining susceptibility to GDM). Inherited abnormalities of pancreas and β cell function may be involved in the etiology of GDM.^25^ In our study fifty-four (72%) of GDM had family history of diabetes. Our findings are consistent with study conducted in Iran.^26^

Family dietary patterns can affect the risk of GDM.As we observed that those who had previous history of GDM were more likely to have GDM p value=0.001. It appears that it may be due to same dietary patterns which the family follows. Other studies have confirmed our findings.^8^,^27^

Maternal age, education, parity, socioeconomic statuses are the factors which determine dietary intake patterns. Studies^28^,^29^ have also shown that older age pregnant women were more likely to eat healthy diet in periconception and during pregnancy but in our study we did not find any association. A study conducted in China^30^ has stated some discrepancies in dietary intake with socioeconomic status. Pregnant women of upper class had more consumption of vegetables, fruit, dairy, soybean and nuts, meat, fish and shrimp, eggs, edible oil, alcohol and dietary variety. This was also not proven by our study.

### Limitations

It includes under reporting of dietary intake on three days recall and FFQ. Some of the participants could not recall and remember the meal timings during the day.

## CONCLUSION

The study concludes that most of our women have followed less healthy dietary intake pattern and this patterns strongly influence the risk of GDM. The most contributing factors to risk of GDM are higher intake of carbohydrate rich diet and lesser consumption of fruits and vegetables.

### Recommendations

Health care providers should counsel and educate women on in expensive, seasonal and healthy food intake during pregnancy. Diet counseling for pregnant women at public hospitals can focus on preventing adverse pregnancy outcomes associated with GDM

### Importance of study

Determining dietary intake patterns in pregnancy will enable health care providers to counsel and educate the pregnant women and their family members the importance of in-expensive, seasonal and healthy food intake during pregnancy. It would help to avoid adverse pregnancy outcomes in relation to GDM.

### Author`s Contribution:

**SN:** Conception, design, and did write up. She is also responsible for the integrity and accuracy of the study. **FA:** Data collection and Interpretation of data. **SR:** Data analysis and contributed in write up. **AK:** Review, Final approval of the version to be published.
